# Cadmium Treatment Alters the Expression of Five Genes at the *Cda1* Locus in Two Soybean Cultivars [*Glycine Max* (L.) Merr]

**DOI:** 10.1155/2014/979750

**Published:** 2014-06-02

**Authors:** Yi Wang, Xue Xiao, Tiequan Zhang, Houyang Kang, Jian Zeng, Xing Fan, Lina Sha, Haiqin Zhang, Kangfu Yu, Yonghong Zhou

**Affiliations:** ^1^Triticeae Research Institute, Sichuan Agricultural University, Wenjiang, Sichuan 611130, China; ^2^Greenhouse and Processing Crops Research Centre, Agriculture and Agri-Food Canada, 2582 County Road 20, Harrow, ON, Canada N0R 1G0; ^3^College of Resources and Environment, Sichuan Agricultural University, Wenjiang, Sichuan 611130, China

## Abstract

Westag 97 has larger capacity of Cd accumulation in roots which prevents Cd from translocating into stems and leaves; conversely, AC Hime has smaller capacity of Cd accumulation in roots; more Cd is transported into stems and leaves. The different capacity of Cd in roots between Westag 97 and AC Hime causes the different Cd concentration in seeds. Meanwhile, according to the different expression levels of RSTK, ISCP, and H^+^-ATPase between Westag 97 and AC Hime, RSTK may be involved in transporting Cd into stems and leaves; H^+^-ATPase may be correlated to the capacity of Cd accumulation in roots; and Cd caused some changes of fundamental life process which leaded to the different expression patterns of ISCP between Westag 97 and AC Hime.

## 1. Introduction

Due to anthropogenic activities, environmental health has been suffering from heavy metal contamination. Cadmium (Cd) is a toxic heavy metal for humans, animals, and plants. In North America, the concentration of Cd in surface soils in the major crop producing regions of the continental United States ranged from 0.005 to 2.400 mg/kg, with mean and median values of 0.270 and 0.200 mg/kg, respectively [1]. Crops absorb Cd from soils and eventually translocate Cd to edible parts [2, 3]. Therefore, consumption, either directly or indirectly, of edible parts with high levels of Cd could be a food safety concern.

At high concentrations Cd can cause severe physiological and morphological damages to plants, such as stunted root and shoot growth [4–6], chlorosis, decreased reproducibility [7], and reduced water and nutrient uptake [8]. Cd stress can affect enzyme activities [5, 9], alter membrane permeability [8], and disrupt cell transport processes [10]. Cd stress can also disturb cellular redox control [11], damage the light harvesting complex II [12] and photosystems I and II [13], and decrease carbon assimilation and chlorophyll content [14].

The expression level of Cd-induced gene or transcription factor has been investigated in many species, which include some Cd-responsive genes and transcription factors [15], several antioxidative enzyme genes [9], a circadian gene,* elongation factor 4* (*ELF4*) [16] in* Arabidopsis thaliana*, a mitogen-activated protein kinase gene in rice [6], several antioxidative enzyme genes in pea [17], Cd detoxification and antioxidant response genes in barley [18], and some metal transporters genes in* Solanum torvum* Sw. cv. Torubamubiga [19]. However, the molecular responses to Cd in soybean have not been reported.

Soybean has long been a staple food for Asians, especially as soymilk, tofu, and oil [20]. Many soybean cultivars can accumulate high Cd concentration in seed when grown on Cd-polluted soil [4, 21]. The Food and Agriculture Organization (FAO) of the World Health Organization (WHO) proposed an upper limit of 0.2 mg/kg Cd concentration in safety soybean seeds [22]. Genetic studies on recombinant inbred line populations have shown that Cd accumulation in soybean seeds is controlled by a major locus* Cda1* on linkage group K [23, 24]. The* GmHMA3* located at the* Cda1* locus was demonstrated to sequester Cd in roots to limit Cd transportation into shoots [25]. Because the regulation of metal homeostasis is complex, other metal-specific transport proteins and metal nonspecific genes may be involved. In our previous study, five metal nonspecific genes, a receptor-like serine/threonine-protein kinase (RSTK, glyma09g06160), a plasma membrane H^+^-transporting ATPase (H^+^-ATPase, glyma09g06250), an iron-sulfur cluster scaffold protein nfu-related (ISCP, glyma09g06300), and two uncharacterized conserved protein (UCP1, glyma09g06220, and UCP2, glyma09g06310), were found at the* Cda1* locus [23].

The objective of this study was to reveal that Westag 97, a low seed Cd accumulator, sequestrates Cd in roots and restricts it from loading into xylem and transporting to leaves and seeds; AC Hime, a high seed Cd accumulator, has a smaller capacity of Cd accumulation in roots, and it translocates and stores more Cd in stems and leaves. Due to different mechanisms of Cd sequestration and translocation between Westag 97 and AC Hime, we investigated the responses of the five genes at the* Cda1* locus to Cd treatment to understand what roles they may play in Cd accumulation in soybean.

## 2. Materials and Methods

### 2.1. Plant Materials and Growth Conditions

Two soybean cultivars, Westag 97 [26], a low Cd accumulator, and AC Hime [27], a high Cd accumulator, were used for this study. The seeds of the two cultivars were from the soybean breeding program at the Greenhouse and Processing Crops Research Centre, Agriculture and Agri-Food Canada. The seeds were sterilized with 5% NaOCl (Cat. number 425044, Sigma) for 5 minutes and rinsed with ddH_2_O three times and then germinated on ddH_2_O soaked filter paper in glass petri plates at room temperature for 7 days (d). The seedlings were grown in nutrient solution with pH 5.7 (Murashige and Skoog basal salt with minimal organics, Sigma). The conditions in the growth chamber were set under 16/8 hours' (h) light/dark periodicity, at 25°C, and 70% humidity. On the 15th d, the plants were cultivated in a nutrient solution that contained 0 *μ*M (control) or 1.6 *μ*M CdSO_4_ (Cat. number 383082, Sigma), and the nutrient solution was refreshed every 5 d. The pH of the nutrient solution and the Cd concentration were adjusted to match those found in soil solutions of Cd-elevated soils [4, 28, 29].

For RNA isolation, leaf, stem, and root samples, each from four plants, were collected at two sampling times (2 and 24 h after Cd treatment) and then snap-frozen in liquid nitrogen and stored at −80°C until RNA extraction. The experiment had a total of 48 samples (two genotypes, three tissues, two treatments, two sampling times, and two biological replications).

For Cd concentration analysis, leaf, stem, and root samples, each from the Cd treated plants, were collected at two sampling times (17th d and 22nd d after the Cd treatment) and then dried for 2 d at 80°C. The experiment had a total of 72 samples (two genotypes, three tissues, two treatments, two sampling times, and three biological replications).

### 2.2. Cadmium Analysis

Dried plant tissues (leaves, stem, and roots) were ground using a high speed (20,000 rpm) mill (Knifetec Sample Mill; Foss, Eden Prairie, MN, USA) to a particle powder (<0.5 mm) in order to determine the Cd concentration. The powder of tissues was digested at 320°C with concentrated sulphuric acid and hydrogen peroxide. Concentrations of Cd in digestions were determined using an atomic absorption spectrometer, Analyst 400 (PerkinElmer, CT, USA). The limit of quantification was 0.006 mg/L for the instrument and 0.016 mg/L for the method. The reference standard solution was purchased from the Fisher Scientific Ltd. (ON, Canada). All data analysis was done with SPSS 20.0 and all figures were drawn with Sigmaplot 12.0.

### 2.3. RNA Isolation and cDNA Synthesis

The UltraClean Plant RNA Isolation Kit (Cat. number 13300-50, Mo Bio Laboratories Inc., CA) was used to extract total RNA. RNA integrity was verified using Experion Automated Electrophoresis Station (Experion starter kit, RNA StdSens regents, Bio-Rad, CA, USA). The RNA concentration was measured three times on the NanoDrop ND-1000 spectrophotometer (NanoDrop Technologies). All RNA samples were stored at −80°C.

Before cDNA synthesis, 3 *μ*g RNA was treated with RQ1 RNase-Free DNase (Cat. number M1601, Promega, CA, USA) to remove contaminated genomic DNA. First strand cDNA was synthesized using Superscript II Reverse Transcriptase (Cat. number 18064-014, Invitrogen, CA, USA) and oligo-dT_(12–18)_ (Invitrogen, CA, USA). The quantity and quality of synthesized cDNA were determined by NanoDrop ND-1000 spectrophotometer. All cDNA was diluted to 6.25 ng/*μ*L and stored at −20°C until quantitative real-time reverse transcription polymerase chain reaction (qRT-PCR).

### 2.4. qRT-PCR Primer Design

Four pairs of primers, actin 3 (ACT3), elongation factor 1B (ELF1B), F-box protein (F-box), and protein phosphatase PP2A regulatory subunit A (PP2A), were obtained from Jian et al. [30] and Wang et al. [31]. The mRNA sequences of five other primers, RSTK, UCP1, H^+^-ATPase, ISCP, and UCP2, were obtained from the Soybase (http://www.soybase.org/). The qRT-PCR primers were designed by Beacon Designer 7.9 (Table 1). The size of the PCR products was verified on a 1.5% agarose gel electrophoresis after qRT-PCR analysis.

### 2.5. qRT-PCR and Normalized Gene Expression

qRT-PCR was performed in a 96-well plate with the CFX-96 real-time system (Bio-Rad, CA, USA). Each reaction of 14 *μ*L contained 25 ng/4 *μ*L cDNA, 1.5 *μ*L (3 pmole/*μ*L) each forward and reverse primer, and 7 *μ*L iQ SYBR Green Supermix (Bio-Rad, CA, USA). Each cDNA sample was amplified in triplicates (three technical replicates). The PCR reaction conditions were 50°C for 2 minutes, 95°C for 10 minutes, 40 cycles of 15 seconds at 95°C, and 1 minute at 60°C, followed by the generation of a dissociation curve by increasing temperature starting from 65 to 95°C to check for specificity of amplification. Baseline and threshold cycle (Ct value) were automatically determined using Bio-Rad CFX manager v1. 6. 541. 1028 with default parameters. ACT3, PP2A, ELF1B, and F-box, which were identified as the best four reference genes for gene expression in soybean exposed to Cd [31], were used as the reference genes. The ^ΔΔ^Ct method was used to evaluate the quantities of each amplified product according to the user manual. All data were analysed with SPSS 20.0, and all figures were drawn with Sigmaplot 12.0.

### 2.6. Validation of Gene Expression Stability

A total of 10 genes, including ACT3, PP2A, ELF1B, F-box, RSTK, H^+^-ATPase UCP1, UCP2, ISCP, and AW152957 (a tag), were used to validate gene expression stability. The software of geNorm V3.5 [32] and NormFinder [33] was used to evaluate the stability of these genes. Expression levels for each sample were expressed relative to the sample with the highest expression. The mean Ct values of three technical replicates were converted into relative quantities and imported into the geNorm V3.5 software and the NormFinder software, respectively.

## 3. Results

### 3.1. Cd Concentration in Tissues

**Figure 1:**
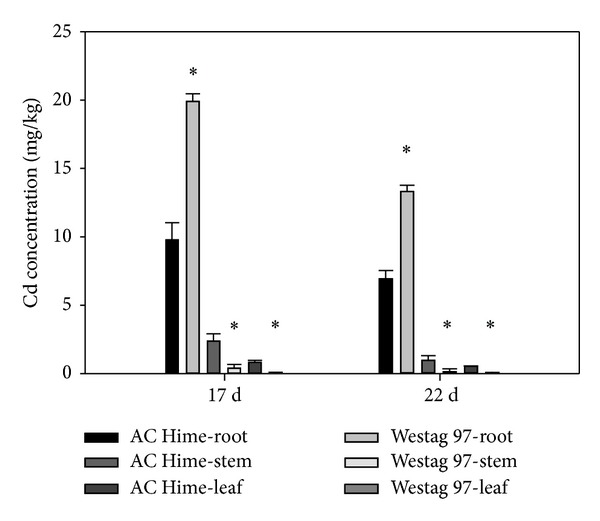
The Cd concentration of three tissues of AC Hime and Westag 97 exposed to 1.6 *μ*M Cd at different times. The bar represents standard error (*n* = 3). The value that is statistically different at *P* < 0.05) is indicated by ∗.

The Cd concentrations of all samples collected from the control (0 *μ*M) treatment were below the detectable limit. As shown in Figure 1, the amount of Cd accumulation in roots of Westag 97 (19.820 ± 0.554 mg/kg) was significantly higher (*P* = 0.0002) than that of AC Hime (9.730 ± 1.253 mg/kg) on the 17th d after Cd treatment. Conversely, the Cd concentration in leaves and stems of AC Hime (0.803 ± 0.164 mg/kg and 2.343 ± 0.541 mg/kg, resp.) was significantly higher (*P* = 0.0014 and 0.0052, resp.) than that in leaves and stems of Westag 97 (0.03 ± 0.011 mg/kg and 0.393 ± 0.272 mg/kg, resp.). Similar results were detected on the 22nd d. The Cd concentration in the roots of Westag 97 (23.277 ± 0.460 mg/kg) was also significantly higher (*P* = 0.00013) than that in roots of AC Hime (6.887 ± 0.611 mg/kg). The Cd concentration in leaves and stems of AC Hime (0.517 ± 0.0151 mg/kg and 0.940 ± 0.330 mg/kg, resp.) was significantly higher (*P* = 0.00014 and 0.0213, resp.) than that in leaves and stems of Westag 97 (0.033 ± 0.0571 mg/kg and 0.117 ± 0.200 mg/kg, resp.).

### 3.2. Gene Expression

**Figure 2:**
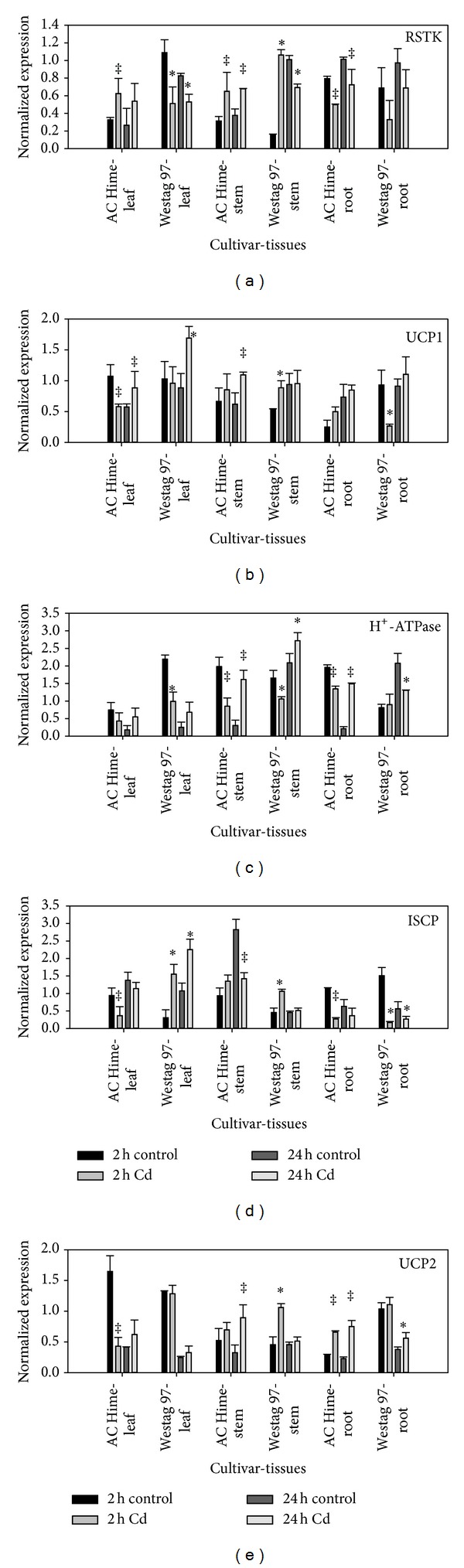
The expression pattern of RSTK, H^+^-ATPase, ISCP, UCP1, and UCP2 in tissues of AC Hime and Westag 97 exposed to different Cd concentrations at different times. Control: 0 *μ*M Cd, High: 1.6 *μ*M Cd, two sampling times at the 2 and 22 h. Each data point is the mean of the two biological replicates with three technical replicates. The bar represents the standard error (*n* = 3). The value that is statistically different at *P* < 0.05 is indicated by ‡ (AC Hime) and ∗ (Westag 97), respectively.

The transcriptional levels of the five genes in both AC Hime and Westag 97 were altered in response to the external Cd treatment (Figure 2).

In AC Hime, Cd significantly upregulated the RSTK expression levels in stems at both sampling times. In Westag 97, Cd significantly upregulated the transcriptional level of RSTK in stems at 2 h, but significant downregulation was observed at 24 h. In leaves, Cd upregulated the expression levels of RSTK in AC Hime at both sampling times, more significantly at 2 h. Conversely, Cd significantly repressed the RSTK gene repression in Westag 97 at both sampling times. Although Cd reduced the expression levels in roots, the expression trend of RSTK in leaves and stems was significantly different between AC Hime and Westag 97 (Figure 2(a)).

In leaves of AC Hime, the expression of UCP1 was significantly decreased at 2 h but increased at 24 h (Figure 2(b)). Significant increase was also observed in leaves of Westag 97 at 24 h. In stems, Cd significantly increased the transcriptional levels of UCP1 in both AC Hime and Westag 97 at 2 h and 24 h, respectively. In roots, Cd increased the expression level of UCP1 in AC Hime at both sampling times, whereas it significantly reduced the expression level in Westag 97 at 2 h.

The transcriptional trends of H^+^-ATPase in both leaves and stems were similar between AC Hime and Westag 97, but the different expression patterns were observed in roots (Figure 2(c)). The transcriptional levels of H^+^-ATPase were reduced in both leaves and stems at 2 h, but increased at 24 h. In roots of AC Hime, the transcriptional levels of H^+^-ATPase were significantly reduced at 2 h but significantly increased at 24 h. In roots of Westag 97, Cd only significantly reduced the transcriptional level of H^+^-ATPase at 24 h.

The transcriptional levels of ISCP in leaves of AC Hime were reduced at both sampling times, but significantly increased in Westag 97 (Figure 2(d)). In stems, Cd significantly reduced the transcriptional level in AC Hime only at 24 h, but it significantly upregulated the transcriptional level in Westag 97 at 2 h. In roots, the expression trend of ISCP in AC Hime was similar to that in Westag 97 at both sampling times.

The effects of Cd on UCP2 expression were not pronounced in Westag 97, except in stems at 2 h and in roots at 24 h (Figure 2(e)). However, pronounced expressional changes were observed in AC Hime. Significant reduction was detected in leaves of AC Hime at 2 h, but significant increases were observed in roots at both sampling times and in stems at 24 h (Figure 2(e)).

### 3.3. Gene Expression Stability

**Figure 3:**
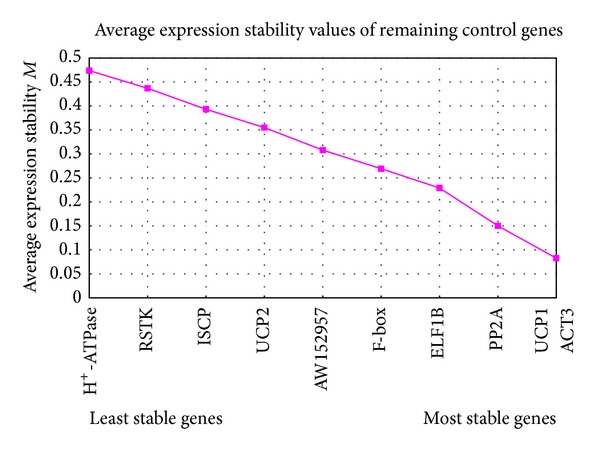
Expression stability (*M*) and ranking of ten candidate reference genes after two soybean cultivars were exposed to different Cd concentrations for two time courses. *M* values were calculated by geNorm following stepwise exclusion of the least stable gene (highest *M* value) across all 24 samples.

The expression stabilities (*M*) of the genes, calculated with the geNorm, indicated that UCP1 and ACT3 were the most stable reference genes, followed by PP2A and ELF1B, with H^+^-ATPase, RSTK, ISCP, and UCP2 as the least stable genes (Figure 3). Similar results were obtained with the NormFinder (see Supplementary Table 1 in Supplementary Material available online at http://dx.doi.org/10.1155/2014/979750). UCP1 was the most stable gene in all 11 tested groups, followed by ACT3. and ATPase, with ISCP as the least stable gene. H^+^-ATPase and ISCP are the least stable genes.

## 4. Discussion

In higher plants, high Cd concentrations have been used in many studies to document the phytotoxic effects and gene transcriptional changes [4–7, 9, 11, 12, 14]. In the present study, although 1.6 *μ*M Cd was not higher than those studies, brown spots on leaves were observed after 3 weeks of Cd treatment (data not shown), which is an indication of the phytotoxic effects of Cd on soybean plants (Morrison, personal communication). Since Cd toxicity varies with growth conditions [18, 34], the Cd concentration used in this study was sufficient for studying Cd uptake and its effects on gene expression.

Cd concentration in soybean seeds was positively correlated to Cd concentration in shoots at the vegetative stage [35]. Shoot Cd concentration is determined by the Cd accumulation capacity of root. Cultivars with a smaller capacity to accumulate Cd in roots would translocate and accumulate more Cd in shoots [21]. In the present study, the Cd concentration in roots of Westag 97 was significantly higher (*P* = 0.0002) than that in roots of AC Hime, but the Cd concentration in leaves and stems of AC Hime was significantly higher (*P* = 0.0014 and 0.0052, resp.) than the Cd concentration in leaves and stems of Westag 97. These results indicated that Westag 97, a low seed Cd accumulator, sequestrated Cd in roots and restricted it from loading into xylem and transporting to leaves and seeds and that AC Hime, a high seed Cd accumulator with a smaller capacity of Cd accumulation in roots, translocated and accumulated more Cd in stems and leaves.

The expression levels of five candidate genes were investigated under Cd treatment to study their responses to Cd stress. Firstly, we evaluated the expression stability of these genes, and found that UCP1 was more stably expressed than other four previously validated reference genes [31]. H^+^-ATPase, RSTK, ISCP, and UCP2 were the least stable genes, which indicated that the expressions of these genes are regulated by Cd.

The RSTK family is involved in signal transduction pathways in plants and interacts with membrane receptor proteins. Previous studies have shown that the expression levels of RSTKs are readily influenced by some biotic/abiotic stresses. For example, the RSTK was downregulated by NaCl stress in a hybrid between* Populus simonii *×* Populus nigra* [36], significantly changed by salt, heat, drought, and high salinity in tobacco [37], up- or downregulated by ripening inhibitor in tomato [38], and downregulated by diseases in* Brachypodium distachyon* [39]. In this study, the expression level of a soybean RSTK at the* Cda1* locus was regulated by Cd. In roots, Cd treatment reduced the expression level of the RSTK more significantly in AC Hime than in Westag 97. In leaves and stems, the Cd-induced expression patterns of RSTK were opposite between AC Hime and Westag 97. The expression levels of RSTK were significantly increased by Cd in AC Hime but were decreased in Westag 97. These results indicated that the RSTK is probably involved in Cd transportation. RSTK can boost Cd transporting into stems and leaves in AC Hime through elevating its expression levels and limit Cd transporting into leaves and stems in Westag 97 through reducing its expression level. Further studies, however, are required to prove this hypothesis.

H^+^-ATPase, the only proton pump operator in plasma membranes, not only regulates the ion homeostasis, but also regulates the growth and development processes in plants [40, 41]. Abiotic stresses, therefore, could change the gene expression of H^+^-ATPase in different species [41–45]. Data concerning Cd action on the H^+^-ATPase are limited and also contradictory. For instance, in roots of* Cucumis sativus*, Cd markedly decreased the expression level of H^+^-ATPase exposed to 100 *μ*M Cd [45], but it increased the expression level of H^+^-ATPase exposed to 10 *μ*M Cd [41], which indicated that the expression pattern of H^+^-ATPase was regulated by different Cd concentration even in the same cultivars. In the present study, although the Cd accumulation capacity differs in leaves and stems between AC Hime and Westag 97, the expression trends of H^+^-ATPase in both leaves and stems of the two cultivars were similar. The expression levels were different in roots between AC Hime and Westag 97, which consisted of different Cd capacity. These results indicated that cultivars' effect on the expression of the soybean H^+^-ATPase exposed to Cd and the soybean H^+^-ATPase is probably involved in Cd transporting to root vacuoles in Westag 97.

ISCPs are ubiquitous and required to sustain fundamental life process of various organisms from lower bacteria to higher eukaryotes [46]. Many studies indicated that they play important roles in electron transfer, substrate binding/activation, iron/sulphur storage, regulation of gene expression, and enzyme activity [46–48]. In the present study, the gene expression levels of ISCP were also regulated by Cd. Cd significantly reduced the gene expression level in roots of both AC Hime and Westag 97. Similar to the RSTK, the expression patterns of ISCP in leaves and stems were opposite between AC Hime and Westag 97, which indicated that Cd caused some changes of fundamental life process; meanwhile, we detected the phytotoxic effects of Cd.

## 5. Conclusion

Westag 97 has larger capacity of Cd accumulation in roots so that prevents Cd from translocating into stems and leaves; conversely, AC Hime has smaller capacity of Cd accumulation in roots; more Cd is transported into stems and leaves. The different capacity of Cd in roots between Westag 97 and AC Hime causes the different Cd concentration in seeds. Meanwhile, according to the different expression levels of RSTK, ISCP, and H^+^-ATPase between Westag 97 and AC Hime, RSTK may be involved in transporting Cd into stems and leaves; H^+^-ATPase may be correlated to the capacity of Cd accumulation in roots; and Cd caused some changes of fundamental life process which led to the different expression patterns of ISCP between Westag 97 and AC Hime.

## Supplementary Material

In order to evident the results calculated from geNome, NormFinder was used to calculate and rank the stability values for these ten genes in each of the 11 groups. UCP1 with the lowest stability value, was the best reference gene in all 11 tested groups, followed by ACT3. H^*+*^- ATPase and ISCP with the highest stability values, were the least stable genes.

## Figures and Tables

**Table 1:** The genes located in original region of major locus *Cda1* or QTL were analyzed for expression pattern.

Loci ID	Annotation	Primer	
Glyma09g06160	Serine-threonine protein kinase, plant type	F	TTCAACGGATGAAAGGAAGG
		R	CAATGCAACACCCAAGAAGA
Glyma09g06220	Uncharacterized conserved protein, contains kelch repeat	F	AGGAGGAGCCATGCTGGAGA
		R	CCAGCAACAGACCCACCAAA
Glyma09g06250	Plasma membrane H^+^-transporting ATPase	F	TACTGATGCGGCAAGAAGTG
		R	AACGCTCAATCCAGGTTCTG
Glyma09g06300	Iron-sulfur cluster scaffold protein nfu-related	F	CCTGGCTTGATGGCTGATG
		R	TGTCCATTATCTGCTCACTT
Glyma09g06310	Uncharacterized conserved protein	F	GCTCAAAGGAAAGGAGTGGAA
		R	ATCTCTCAAGGGCAGTGTAGG
