# Supplementary material for: Cadmium Treatment Alters the Expression of Five Genes at the Cda1 Locus in Two Soybean Cultivars [Glycine Max (L.) Merr]
Source: ScientificWorldJournal. 2014 Jun 2;2014:979750. doi: 10.1155/2014/979750 (PMC4060588; doi:10.1155/2014/979750)
Supplement: Supplementary file 1 — In order to evident the results calculated from geNome, NormFinder was used to calculate and rank the stability values for these ten genes in each of the 11 groups. UCP1 with the lowest stability value, was the best reference gene in all 11 tested groups, followed by ACT3. H+- ATPase and ISCP with the highest stability values, were the least stable genes. [file 979750.f1.pdf]

Supplementary Table 1. Ranking of these genes in total samples according to their stability value using NormFinder

	ACT3		RSTK		H^+-ATPase		AW152957		UCP1		ELF1B		F-Box		PP2A		ISCP		UCP2		Best Gene	Best combination of two genes
Groups	R	Sv	R	Sv	R	Sv	R	Sv	R	Sv	R	Sv	R	Sv	R	Sv	R	Sv	R	Sv		
Total	2	0.465	7	1.088	10	2.669	3	0.528	1	0.415	6	0.735	4	0.595	5	0.632	9	2.362	8	2.218	UCP1	
Cultivars	2	0.090	7	0.219	10	0.546	4	0.105	1	0.081	6	0.142	5	0.122	3	0.104	9	0.484	8	0.458	UCP1	ACT3/ UCP1
Cd treatments	2	0.094	7	0.221	10	0.550	3	0.107	1	0.074	6	0.135	5	0.122	4	0.109	9	0.487	8	0.453	UCP1	ACT3/ UCP1
Times	2	0.266	7	0.465	10	0.855	5	0.412	1	0.236	3	0.337	4	0.369	6	0.421	9	0.755	8	0.726	UCP1	ACT3/ UCP1
Tissues	2	0.356	7	0.889	10	2.090	5	0.468	1	0.291	6	0.518	3	0.405	4	0.417	9	2.062	8	1.757	UCP1	ACT3/ UCP1
Cultivars-Cd	2	0.108	7	0.307	10	0.787	4	0.136	1	0.104	6	0.174	5	0.165	3	0.132	9	0.700	8	0.653	UCP1	ACT3/ UCP1
Cultivars-times	2	0.127	7	0.397	10	1.099	3	0.135	1	0.115	6	0.205	4	0.184	5	0.195	9	1.007	8	0.925	UCP1	ACT3/ UCP1
Cultivars-tissues	2	0.495	7	0.990	10	2.153	5	0.527	1	0.403	6	0.606	4	0.524	3	0.510	9	2.088	8	1.808	UCP1	ACT3/ UCP1
Cultivars-Cd-times	2	0.143	7	0.540	10	1.668	3	0.193	1	0.124	6	0.233	5	0.220	4	0.198	9	1.522	8	1.411	UCP1	ACT3/ UCP1
Cultivars-Cd-tissues	2	0.568	7	1.094	10	2.154	4	0.664	1	0.546	6	0.723	5	0.683	3	0.613	9	2.131	8	1.854	UCP1	ACT3/PP2A
Cultivars-times-tissues	2	0.674	7	1.314	10	2.284	6	0.796	1	0.636	5	0.771	4	0.715	3	0.695	9	2.163	8	1.973	UCP1	ACT3/UCP1

Note: R: Rank; SV: Stability Value
